# RETRACTION: The Effect of Preoperative Smoking and Smoke Cessation on Wound Healing and Infection in Post‐Surgery Subjects: A Meta‐Analysis

**DOI:** 10.1111/iwj.70421

**Published:** 2025-03-26

## Abstract

**RETRACTION:** LiuD. , ZhuL. , and YangC. , “The Effect of Preoperative Smoking and Smoke Cessation on Wound Healing and Infection in Post‐Surgery Subjects: A Meta‐Analysis,” International Wound Journal 19, no. 8 (2022): 2101–2106, 10.1111/iwj.13815.35451193 PMC9705191

The above article, published online on 22 April 2022, in Wiley Online Library (http://onlinelibrary.wiley.com/), has been retracted by agreement between the journal Editor in Chief, Professor Keith Harding; and John Wiley & Sons Ltd. Following an investigation by the publisher, all parties have concluded that this article was accepted solely on the basis of a compromised peer review process. The editors have therefore decided to retract the article. The authors did not respond to our notice regarding the retraction.

**RETRACTION:** D. Liu , L. Zhu , and C. Yang , “The Effect of Preoperative Smoking and Smoke Cessation on Wound Healing and Infection in Post‐Surgery Subjects: A Meta‐Analysis,” International Wound Journal 19, no. 8 (2022): 2101–2106, 10.1111/iwj.13815.35451193 PMC9705191

The above article, published online on 22 April 2022, in Wiley Online Library (http://onlinelibrary.wiley.com/), has been retracted by agreement between the journal Editor in Chief, Professor Keith Harding; and John Wiley & Sons Ltd. Following an investigation by the publisher, all parties have concluded that this article was accepted solely on the basis of a compromised peer review process. The editors have therefore decided to retract the article. The authors did not respond to our notice regarding the retraction.

